# Supplementary material for: COZOID: contact zone identifier for visual analysis of protein-protein interactions
Source: BMC Bioinformatics. 2018 Apr 6;19:125. doi: 10.1186/s12859-018-2113-6 (PMC5889581; doi:10.1186/s12859-018-2113-6)
Supplement: Supplementary file 2 — Software build. Executable binary file of the software tool. (ZIP 118,784 kb) [file 12859_2018_2113_MOESM2_ESM.zip › cozoid/java/docs/beansbinding-1.2.1-doc.zip!/org/jdesktop/swingbinding/impl/AbstractColumnBinding.html]

AbstractColumnBinding (Beans Binding)


---


|  |  |  |
| --- | --- | --- |
| **Overview** \| **Package** \| **Class** \| **Use** \| **Tree** \| **Deprecated** \| **Index** \| **Help** | |  |
| PREV CLASS   **NEXT CLASS** | **FRAMES**    **NO FRAMES**     **All Classes** |  |
| SUMMARY: NESTED \| FIELD \| CONSTR \| METHOD | DETAIL: FIELD \| CONSTR \| METHOD |  |


---


## org.jdesktop.swingbinding.impl Class AbstractColumnBinding

```
java.lang.Object
  org.jdesktop.beansbinding.Binding
      org.jdesktop.swingbinding.impl.AbstractColumnBinding
```

**Direct Known Subclasses:**: JListBinding.DetailBinding, JTableBinding.ColumnBinding

---

``` public abstract class AbstractColumnBinding extends Binding ```

---

**Nested Class Summary**

**Nested classes/interfaces inherited from class org.jdesktop.beansbinding.Binding**

`Binding.SyncFailure, Binding.SyncFailureType, Binding.ValueResult<V>`


**Constructor Summary**

`AbstractColumnBinding(int column,
Property columnSource,
Property columnTarget,
java.lang.String name)`


| **Method Summary** | |
| --- | --- |
| `void` | `bindImpl()`             Called by `Binding.bind()` to allow subclasses to initiate binding. |
| `int` | `getColumn()` |
| `protected  void` | `setColumn(int column)` |
| `void` | `unbindImpl()`             Called by `Binding.unbind()` to allow subclasses to uninitiate binding. |

**Methods inherited from class org.jdesktop.beansbinding.Binding**

`addBindingListener, addPropertyChangeListener, addPropertyChangeListener, bind, bindUnmanaged, firePropertyChange, getBindingListeners, getConverter, getName, getPropertyChangeListeners, getPropertyChangeListeners, getSourceNullValue, getSourceObject, getSourceProperty, getSourceUnreadableValue, getSourceValueForTarget, getTargetNullValue, getTargetObject, getTargetProperty, getTargetValueForSource, getValidator, isBound, isManaged, isSourceUnreadableValueSet, notifySynced, notifySyncFailed, paramString, refresh, refreshAndNotify, refreshAndNotifyUnmanaged, refreshUnmanaged, removeBindingListener, removePropertyChangeListener, removePropertyChangeListener, save, saveAndNotify, saveAndNotifyUnmanaged, saveUnmanaged, setConverter, setManaged, setSourceNullValue, setSourceObject, setSourceObjectUnmanaged, setSourceProperty, setSourceUnreadableValue, setTargetNullValue, setTargetObject, setTargetObjectUnmanaged, setTargetProperty, setValidator, sourceChangedImpl, targetChangedImpl, throwIfBound, throwIfManaged, throwIfUnbound, toString, unbind, unbindUnmanaged, unsetSourceUnreadableValue`

**Methods inherited from class java.lang.Object**

`clone, equals, finalize, getClass, hashCode, notify, notifyAll, wait, wait, wait`

**Constructor Detail**

### AbstractColumnBinding

```
public AbstractColumnBinding(int column,
                             Property columnSource,
                             Property columnTarget,
                             java.lang.String name)
```


**Method Detail**

### getColumn

```
public final int getColumn()
```

---


### setColumn

```
protected final void setColumn(int column)
```

---


### bindImpl

```
public void bindImpl()
```

:   **Description copied from class: `Binding`**
:   Called by `Binding.bind()` to allow subclasses to initiate binding.
    Subclasses typically need not install `PropertyStateListeners`
    on the source property and target property as they will be notified
    by calls to `Binding.sourceChangedImpl(org.jdesktop.beansbinding.PropertyStateEvent)` and `Binding.targetChangedImpl(org.jdesktop.beansbinding.PropertyStateEvent)`
    when the source and target properties change respectively.

    :   **Specified by:**: `bindImpl` in class `Binding`
    :   **See Also:**: `Binding.unbindImpl()`

---


### unbindImpl

```
public void unbindImpl()
```

:   **Description copied from class: `Binding`**
:   Called by `Binding.unbind()` to allow subclasses to uninitiate binding.

    :   **Specified by:**: `unbindImpl` in class `Binding`
    :   **See Also:**: `Binding.bindImpl()`


---


|  |  |  |
| --- | --- | --- |
| **Overview** \| **Package** \| **Class** \| **Use** \| **Tree** \| **Deprecated** \| **Index** \| **Help** | |  |
| PREV CLASS   **NEXT CLASS** | **FRAMES**    **NO FRAMES**     **All Classes** |  |
| SUMMARY: NESTED \| FIELD \| CONSTR \| METHOD | DETAIL: FIELD \| CONSTR \| METHOD |  |


---
